# Relationship between Vitamin D Receptor gene polymorphisms and the components of metabolic syndrome

**DOI:** 10.1186/1475-2891-12-96

**Published:** 2013-07-15

**Authors:** Natielen Jacques Schuch, Vivian Cristina Garcia, Sandra Roberta Gouvea Ferreira Vívolo, Lígia Araújo Martini

**Affiliations:** 1 Nutrition Department, School of Public Health, University of São Paulo, Av. Dr. Arnaldo, 715, São Paulo, SP CEP 01246-904, Brazil

**Keywords:** Vitamin D, Vitamin D Receptor gene, Polymorphisms, Parathyroid hormone, Metabolic syndrome

## Abstract

**Background:**

The Vitamin D Receptor gene (VDR) is expressed in many tissues and modulates the expression of several other genes. The purpose of this study was to investigate the association between metabolic syndrome (MetSyn) with the presence of VDR 2228570 C > T and VDR 1544410 A > G polymorphisms in Brazilian adults.

**Methods:**

Two hundred forty three (243) individuals were included in a cross-sectional study. MetSyn was classified using the criteria proposed by National Cholesterol Educational Program - Adult Treatment Panel III. Insulin resistance and β cell secretion were estimated by the mathematical models of HOMA IR and β, respectively. The VDR 2228570 C > T and VDR 1544410 A > G polymorphisms were detected by enzymatic digestion and confirmed by allele specific PCR or amplification of refractory mutation.

**Results:**

Individuals with MetSyn and heterozygosis for VDR 2228570 C > T have higher concentrations of iPTH and HOMA β than those without this polymorphism, and subjects with recessive homozygosis for the same polymorphisms presented higher insulin resistance than those with the heterozygous genotype. There is no association among VDR 1544410 A > G and components of MetSyn, HOMA IR and β, serum vitamin D (25(OH)D_3_) and intact parathormone (iPTH) levels in patients with MetSyn. A significant lower concentration of 25(OH)D_3_ was observed only in individuals without MetSyn in the VDR 1544410 A > G genotype. Additionally, individuals without MetSyn and heterozygosis for VDR 2228570 C > T presented higher concentration of triglycerides and lower HDL than those without this polymorphism.

**Conclusions:**

Using two common VDR polymorphism data suggests they may influence insulin secretion, insulin resistance an serum HDL-cholesterol in our highly heterogeneous population. Whether VDR polymorphism may influence the severity of MetSyn component disorder, warrants examination in larger cohorts used for genome-wide association studies.

## Background

The role of vitamin D for the development and maintenance of bone tissue, as well as for maintaining normal homeostasis of calcium and phosphorus is well established. [1,2]. Currently, the involvement of vitamin D in the components of metabolic syndrome has been suggested [3]. MetSyn is defined as a combination of medical disorders that, when occurring together, increase the risk of developing cardiovascular disease and type 2 diabetes mellitus (T2DM). Although the mechanisms underlying the role of vitamin D in MetSyn remain incompletely explained, it was showed that 25(OH)D_3_ could be related to the occurrence of reduced insulin secretion and sensitivity [4,5], obesity [6,7], diabetes [8-10], and hypertension [11]. Additionally, non-alcoholic fatty liver disease has been suggested as an independent component of MetSyn [12] and a low concentration of 25(OH)D_3_ might play a role in the development and progression of such condition [13].

Activated vitamin D exerts its cellular functions by modulating the transcription of target genes after binding to the nuclear Vitamin D Receptor (VDR) [14]. Several polymorphisms have been reported for the VDR gene such as ApaI (VDR 7975232 C > T), BsmI (VDR 1544410 A > G), FokI (VDR 2228570 C > T), and TaqI (VDR 731236 T > C). Additionally, it has been demonstrated that some of these polymorphisms are associated with type 2 diabetes mellitus, insulin secretion [15,16], as well as with metabolic changes related to obesity [14]. Besides the molecular evidence for the putative relation between VDR polymorphism and such disorders remains unclear, it has been demonstrated that a length of the VDR, affected by the presence of the polymorphisms, could lead a lower activation of target cells, since a longer VDR protein appears to have a decreased transcriptional activity [17].

Although the relationship between vitamin D insufficiency and components of MetSyn has been previously demonstrated [3,18], few studies have examined VDR gene polymorphisms for associations with the risk of these disorders [14,19,20]. Despite the high incidence of year-round sunlight in Brazil, hypovitaminosis D has been reported among different population groups including teenagers, adult, and elderly [21-24]. On the other hand, studies estimate that vitamin D concentrations have a heritability of up to 53% [25], which could be different in our population considering that we have a genetically heterogeneous population.

Thus, this study was designed to investigate the association of components of MetSyn with the presence of polymorphisms in VDR gene [VDR 1544410 A > G (BsmI); VDR 2228570 C > T (FokI)] in Brazilian adults and has demonstrated associations between VDR gene polymorphisms with insulin secretion, insulin resistance and HDL-cholesterol, suggesting that these polymorphisms can affect metabolic syndrome phenotype.

## Methods

### Subjects, anthropometric measurements, and blood pressure

This was a cross sectional study performed between August 2007 and January 2009 in the city of São Paulo, Brazil. Initially, 462 individuals from diabetes mellitus prevention campaigns coordinated by the Primary Healthcare Unit at the School of Public Health, University of São Paulo, and from the Adult Health Survey of the City of São Paulo were invited to participate. Exclusion criteria were: the presence of chronic illnesses that potentially alter vitamin D metabolism, the use of medications that affect bone metabolism, afro-descendant people, pregnant or breastfeeding women, and the use of vitamin supplements. After the selection and removal of individuals who did not accept participation, 243 blood samples were collected for laboratory measurements and genotyping. A signed consent form was obtained from all participants and the ethics committee of the School of Public Health from University of São Paulo approved the study protocol.

Height was measured using a fixed stadiometer with a vertical backboard and movable headboard, with subjects standing on the floor. Weight was taken by asking each individual to stand at the center of the platform of a Tanita™ digital scale (Tanita Corporation of America Inc., Illinois, USA). Body mass index [BMI = weight(kg)/height(m)^2^] was calculated. Waist circumference was measured while the subjects were standing up, with a tape placed at the midpoint level between the lower intercostal border and the anterior superior iliac supine while the subject was gently exhaling. Blood pressure was obtained using an automatic blood pressure monitor (Omron model HEM-712C, Omron Health Care, Inc., USA). Three measures were taken at rest in a sitting position, with intervals of five minutes between the measurements. The average from the measurements was taken for analysis. The subjects were also asked about their use of sunscreen and practice of outdoor physical activities. Participants were further divided into two groups, with and without MetSyn, according to criteria adopted by NCEP – ATP III. Briefly, waist circumference ≥ 102 cm for men, ≥ 88 cm for women; fasting glucose ≥ 110 mg/dl; triglycerides ≥ 150 mg/dl; HDL-cholesterol < 40 g/dl for men, <50 mg/dl for women; blood pressure systolic ≥ 130 mm Hg or diastolic ≥ 85 mmHg [26].

### Biochemical analyses of blood samples

Blood samples were obtained by venipuncture after an overnight fast and immediately centrifuged, aliquoted and frozen at -80°C until the beginning of analyses. Serum levels of 25(OH)D_3_ were measured by High Performance Liquid Chromatography (HPLC; Immundiagnostik AG, Germany). Serum levels of intact parathyroid hormone (iPTH) were measured by a electrochemiluminescence assay (Roche Diagnostics™, Brazil), in which, intra and interassay CVs were both 6% with a normal range of 10-65 pg/ml. Serum total levels of calcium were measured by colorimetrical assay (Bioclin™, Brazil), normal range 8.6-10.2 mg/dl. Fasting glucose, total cholesterol and fractions, and triglycerides were determined by enzymatic colorimetric assay (Celm™, Brazil). For these variables, the cut-offs for diagnosis of MetSyn used were those of the NCEP-ATP III [26]. Moreover, plasma insulin was assayed by chemiluminescence (IMMUNOLITE® commercial kit, Siemens Health Diagnostics, USA), and insulin resistance was estimated by the homeostatis model assessment of insulin resistance (HOMA-IR), by the following equation:

$$\mathrm{HOMA}-\mathrm{IR}=\left(\mathrm{fasting}\;\mathrm{glucose}\;X\;\mathrm{fasting}\;\mathrm{insulin}\right)/22.5$$

where fasting glucose and fasting insulin are described in nmol/l and mU/ml, respectively [26]. In addition, HOMA-β-cell function was also calculated by the following formula:

$$\mathrm{HOMA}-\beta =\left(20\;X\;\mathrm{fasting}\;\mathrm{insulin}\right)/\left(\mathrm{fasting}\;\mathrm{glucose}–3.5\right)$$

where fasting insulin and fasting glucose are described in μU/ml and mmol/L, respectively [27].

### Genotyping

Genomic DNA was extracted from the whole blood using the salting out method [28]. After extraction, the quality of the extracted material was visualized in 1% agarose gel and the concentration was obtained by a spectrophotometer Nanodrop 8000 (Thermo Scientific, USA). Genotyping of BsmI (VDR 1544410 A > G) and FokI (VDR 2228570 C > T) polymorphisms in the VDR gene was performed using a technique known as allele specific polymerase chain reaction (ASPCR) or amplification refractory mutation (ARMs) [29]. Detection of the products was made using fluorescence resonance energy. PCRs were carried out with ArrayTape instrumentation, and allele calls were generated based on the clustering of fluorescent signals. Only genotypes with a level of confidence ≥90% were included in the analysis.

### Statistical analysis

Descriptive data were expressed as mean and standard deviation. Normality of the distribution of each variable was analyzed by Kolmogorov-Smirnov test. Logarithmic transformation was performed to obtain a normal distribution for skewed variables (iPTH, BMI, waist circumference, HOMA IR, HOMA β, triglycerides, and HDL-cholesterol). One-way analysis of variance (ANOVA) was used to examine the variation in different MetSyn variables with the genotypes and for adjustment according to age. Differences between the genotypes were assessed by using the Bonferroni LSD test (post-hoc test). Allelic frequencies were estimated by gene counting and genotypic distribution of polymorphisms, and tested for Hardy-Weinberg equilibrium (HWE), by Chi-square analysis. HWE assumes a stable population of adequate size without selective pressures and is used in human genetic studies as a guide to data quality by comparing observed genotype frequencies to those expected within a population. Logistic regression analysis was performed to evaluate associations between clinical and genetic variables. Gender, age, elevated waist circumference, increased glucose fasting levels, presence of arterial hypertension, increased triglycerides levels, decreased HDL cholesterol, increased total cholesterol levels, presence of MetSyn, HOMA IR and β, increased iPTH, and 25(OH)D_3_ levels were included as independent variables, and the presence or absence of VDR gene polymorphisms was characterized as categorical dependent variables. Analyses were conducted with Package for the Social Science SPSS software for Windows, version 18 (SPSS Inc., USA). Statistical significance was assumed when p values were < 0.05.

## Results

### General characteristics of study population

The participants mean age was 51 (±15) years and BMI 26 (± 6) kg/m^2^. Thirty nine percent was male and 61% female. MetSyn was present in 48% of the population. As expected, BMI, waist circumference, blood pressure, fasting glucose, total cholesterol and LDL-cholesterol were significantly higher in individuals with MetSyn, except for HDL cholesterol (44 ±12 vs 41 ±11 mg/dl, p < 0.000). Mean study population vitamin D status was insufficient (22.8 ng/ml), the mean of 25(OH)D_3_ was significantly lower among people without MetSyn (22.8 ± 7.2 vs 24.4 ± 7.6 ng/ml, p = 0.003 respectively). No significant differences were observed for iPTH and serum levels of calcium between the groups with and without MetSyn.

### Distribution of VDR 1544410 A > G (BsmI) and VDR 2228570 C > T (FokI) gene polymorphisms

Following the genotyping the distribution of both VDR BsmI and VDR FokI polymorphisms was performed in those with or without MetSyn. Table 1 shows the frequency of these polymorphisms. Besides the lower frequency of normal dominant homozygous genotype FF and also BB among individuals without MetSyn, there is no difference in genotype frequencies for either polymorphisms, thus demonstrating Hardy-Weinberg equilibrium.

**Table 1:** Genotype distribution of VDR gene polymorphisms among individuals with and without MetSyn

**Genotype**		**With MetSyn**	**Without MetSyn**	**p-value**
**Distribution (%)**		**n = 116**	**n = 127**	
**FokI**	FF	13	8	
(VDR 2228570 C > T)	Ff	47	57	
	ff	40	35	p = 0.16
**BsmI**	BB	20	9	
(VDR 1544410 A > G)	Bb	43	41	
	bb	37	50	p = 0.79

**Legend:** the distribution of the VDR polymorphisms is in Hardy-Weinberg equilibrium. Normal dominant homozygous genotype “FF, BB”; heterozygous genotype “Ff, Bb”; mutant recessive homozygous genotype “ff, bb”.

### VDR 1544410 A > G (BsmI) and VDR 2228570 C > T (FokI) gene polymorphisms and the findings for variables used to demonstrate the presence or absence of metabolic syndrome

The associations among VDR gene polymorphisms, vitamin D status, and the components of MetSyn were evaluated separately according to the presence or absence of MetSyn.

Table 2 presents the distribution of all clinical variables according to the genotypes observed in individuals with MetSyn. No association between VDR BsmI polymorphism was observed with the components of MetSyn, 25(OH)D_3_, or iPTH. However, individuals carrying the mutant recessive homozygous ff genotype presented a significantly higher insulin resistance index (HOMA-IR) than individuals with the heterozygous Ff genotype. Additionally, the presence of at least one f for the VDR FokI polymorphism was associated with higher β-cell secretion (HOMA-β) and iPTH concentrations, them were seen in those with the F allele.

**Table 2:** Mean values of all variables by VDR genotype in subjects with MetSyn

**Variables**	**VDR 2228570 C > T (Fok I)**			**p**	**VDR 1544410 A > G (Bsm I)**			**p**
	**FF**	**Ff**	**ff**		**BB**	**Bb**	**bb**	
	**n = 130**	**n = 91**	**n = 22**		**n = 109**	**n = 102**	**n = 32**	
WC (cm)	103 (±13)	103 (±12)	106 (±13)	0.433	104 (±13)	103 (±13)	104 (±13)	0.863
Triglycerides (mg/dl)	155 (±59)	174 (±81)	163 (±103)	0.204	151 (±59)	173 (±87)	163 (±64)	0.523
BPS (mmHg)	140 (±18)	137 (±16)	147 (±24)	0.377	139 (±14)	139 (±20)	134 (±20)	0.490
BPD (mmHg)	84 (±12)	82 (±10)	85 (±12)	0.726	84 (±10)	83 (±10)	82 (±8)	0.822
Fasting glucose (mg/dl)	99 (±10)	98 (±13)	99 (±14)	0.707	100 (±12)	97 (±13)	99 (±12)	0.404
HDL cholesterol (mg/dl)	43 (±12)	40 (±9)	40 (±12)	0.306	43 (±12)	42 (±10)	40 (±12)	0.231
HOMA IR	2 (±2)	3 (±2)	4 (±3)†	0.014	3 (±1)	3 (±2)	4 (±3)	0.259
HOMA β	100 (±77)	159 (±137)‡	145 (±61)	0.010	123 (±77)	142 (±137)	137 (±107)	0.729
25(OH)D_3_ (ng/ml)	24 (±6.8)	24.8 (±16.8)	24 (±8)	0.828	24 (±6.4)	62 (±8.4)	24 (±8)	0.389
iPTH (pg/dl)	37 (±19)	49 (±22)‡	42 (±25)	0.022	44 (±15)	49 (±22)	46 (±25)	0.649

**Legend:***WC*, Waist circumference; *BPS*, Systolic blood pressure; *BPD*, Diastolic blood pressure; *HOMA IR*, Insulin resistance; *HOMA β*, Cell function; *iPTH*, Intact parathyroid hormone; *25(OH)D*_*3*_, Vitamin D. Data are expressed as mean (Standard Deviation). P values were calculated using ANOVA followed by Bonferroni test and adjusted for age. † significant for the heterozygous genotype; ‡ significant for the normal dominant homozygous genotype.

Then, the same analyses were performed for the group of individuals without MetSyn. These results are present in Table 3. In addition, higher serum triglycerides and lower HDL concentrations were seen in subjects with both the F and f FokI polymorphism. Whilst bb homozygous for the BsmI VDR variant was associated with lower 25(OH)D_3_ values than seen in others. Additionally, the values of diastolic and systolic blood pressure, fasting glucose and waist circumference were very similar among all participants, independently of presence or absence of both VDR BsmI and FokI polymorphisms.

**Table 3:** Mean values of all variables by VDR genotype in subjects without MetSyn

**Variables**	**VDR 2228570 C > T (Fok I)**			**p**	**VDR 1544410 A > G (Bsm I)**			**p**
	**FF**	**Ff**	**ff**		**BB**	**Bb**	**bb**	
	**n = 130**	**n = 91**	**n = 22**		**n = 109**	**n = 102**	**n = 32**	
WC (cm)	89 (±10)	95 (±16)	90 (±9)	0.086	93 (±14)	93 (±13)	86 (±11)	0.293
Triglycerides (mg/dl)	90 (±28)	117 (±54)‡	134 (±80)	0.005	107 (±46)	113 (±58)	100 (±45)	0.696
BPS (mmHg)	122 (±17)	121 (±21)	124 (±14)	0.923	119 (±21)	125 (±16)	121 (±21)	0.194
BPD (mmHg)	75 (±10)	76 (±14)	79 (±10)	0.316	75 (±13)	77 (±11)	74 (±14)	0.556
Fasting glucose (mg/dl)	92 (±10)	92 (±11)	89 (±10)	0.607	92 (±11)	90 (±9)	93 (±8)	0.588
HDL cholesterol (mg/dl)	50 (±11)	45 (±12)‡	42 (±11)	0.031	47 (±13)	47 (±11)	42 (±6)	0.253
Homa IR	1.6 (±1.4)	1.7 (±1.4)	1.0 (±0.4)	0.398	1.7 (±1.7)	1.6 (±0.9)	1.2 (±0.7)	0.749
Homa β	92 (±62)	103 (±80)	87 (±93)	0.711	96 (±81)	107 (±71)	70 (±46)	0.424
25(OH)D_3_ (ng/ml)	20 (±6)	22 (±6.4)	24.4 (±5)	0.062	20.8 (±6)	23 (±6.4)	18 (±7.2)†	0.023
iPTH (pg/dl)	41 (±24)	36 (±18)	38 (±11)	0.019	40 (±20)	37 (±20)	32 (±24)	0.361

**Legend:***WC*, Waist circumference; *BPS*, Systolic blood pressure; *BPD*, Diastolic blood pressure; *HOMA IR*, Insulin resistance; *HOMA β*, Cell function; *iPTH*, Intact parathyroid hormone; 25(OH)D_3_, Vitamin D. Data are expressed as mean (Standard Deviation). P values were calculated using ANOVA followed by Bonferroni test and adjusted for age. † significant for the heterozygous genotype; ‡ significant for the normal dominant homozygous genotype.

Finally, data for all subjects was used for multiple logistic regression to determine associations of VDR polymorphism with MetSyn and its components - Table 4. HDL-cholesterol showed variation with FokI polymorphism (odds ratio, 0.967, β = -0.032; 95% CI = 0.945-0.993; p = 0.01), but no other MetSyn component varied with VDR genotype.

**Table 4:** Logistic regression of traits associated with the presence of VDR 2228570 C > T (Fok I) polymorphism in the study population

**Variables**	**B**	***Odds ratio***	**p**	**95% confidence interval**	
				**Lower**	**Upper**
Age	0.014	1.014	0.244	0.991	1.037
Gender	-0.395	0.674	0.357	0.291	1.561
WC	0.007	1.007	0.662	0.977	1.037
Blood pressure	0.009	1.009	0.594	0.977	1.042
Glucose fasting	0.041	1.041	0.136	0.987	1.099
Triglycerides	0.005	1.005	0.187	0.998	1.012
HDL cholesterol	-0.032	.967	0.011	0.945	0.993
Total cholesterol	0.005	1.005	0.310	0.996	1.014
MetSyn	-0.136	0.474	0.239	0.696	1.095
HOMA IR	-0.128	0.880	0.558	0.573	1.351
HOMA β	0.007	1.007	0.112	0.998	1.017
iPTH	0.000	1.000	0.970	0.985	1.016
25(OH)D_3_	0.007	1.007	0.536	0.985	1.029

**Legend:***WC*, Waist circumference; *MetSyn*, Metabolic syndrome; *HOMA IR* Insulin resistance; *HOMA β*, cell function; *iPTH*, Intact parathyroid hormone; 25(OH)D_3_, Vitamin D.

## Discussion

Our findings suggest that VDR gene polymorphisms may influence the severity of MetSyn component disorder. In individuals with MetSyn, the VDR 2228570 C > T (FokI) polymorphism appeared to be associated with insulin sensitivity and iPTH concentration. Additionally, in individuals without MetSyn, this polymorphism was associated with higher triglycerides and lower HDL levels. Furthermore, when considering all individuals, HDL cholesterol varied independently with VDR FokI polymorphism. In support of the findings, Oh and Barrett-Connor (2002) investigating VDR gene polymorphisms in individuals with type 2 diabetes in the Rancho Bernando Cohort, observed that individuals with the mutant recessive homozygous genotype, the VDR 1544410 A > G (BsmI) polymorphism, presented significantly higher levels of HOMA-IR compared to those with heterozygous or normal homozygous genotypes [20].

Since the early 1980’s, it has been demonstrated that pancreatic insulin secretion is inhibited by vitamin D deficiency, suggesting a role for this vitamin in the regulation of endocrine pancreatic function, especially in the β cell [30]. In addition, the non-genomic pathway dependent on membrane VDR protein promotes rapid calcium fluxes important in inducing effects of vitamin D on insulin release [31-34]. It is already known that 1,25(OH)_2_D_3_ directly influences β cell insulin secretion through the induction of increases in intracellular free calcium concentration through voltage-dependent Ca^2+^ channels [35]. Moreover, though glycemia is a major major characteristic of diabetes mellitus type 2, no association was found between fasting glycemia and VDR gene polymorphisms evaluated in this cohort. Similarly, Malecki and coworkers (2003) have investigated BsmI, TaqI, and FokI VDR gene polymorphisms in 548 Polish individuals with and without T2DM and did not found any association of glycemia with any of the VDR gene polymorphisms either [36].

On the other hand, the association of MetSyn with increased cardiovascular disease may be modulated by non-bony effects of vitamin D such as those on lipid profiles. This possibility is supported by an investigation on whether lack of vitamin D has effect on cholesterol metabolism where serum concentrations of HDL cholesterol was found to be 22% higher in VDR knockout compared to wild type mice [37]. In contrast, different results were observed in humans in the NHANES 2000–2004 survey, which demonstrated that vitamin D deficiency (< 15 ng/mL) was associated with lower concentrations of HDL cholesterol in adults [38]. Results of this study corroborate the latter study, in suggesting that VDR gene polymorphism associated with lower serum 25(OH)D_3_[24], may be linked to lower HDL-cholesterol in adults_._

There are three mechanisms by which our data suggests that the vitamin D-VDR axis could affect lipid profiles. First: Vitamin D induced suppression of PTH secretion, and it has been reported that PTH could reduce lipolysis [39]. Second: Vitamin D increases intestinal calcium absorption and this can trigger a decrease in serum triglycerides levels by reducing the hepatic triglyceride formation and secretion [40], and Third: Vitamin D might improve insulin secretion and insulin sensitivity, thereby indirectly influencing lipid metabolism [41].

In the present study, important associations were also identified for both VDR 2228570 C > T (FokI) or VDR 1544410 A > G (BsmI) polymorphism in individuals without MetSyn. The higher triglycerides and lower HDL in heterozygous VDR 2228570 C > T (FokI) as well lower 25(OH)D_3_ in homozygous recessive VDR 1544410 A > G (BsmI), could be related to the overweight/obese condition of the individuals. There are likely to be many genetic factors in addition to the VDR 1544410 A > G and VDR 2228570 C > T gene polymorphisms that are involved in determination of MetSyn risk. Despite this our data suggests that VDR polymorphisms may contribute to MetSyn component severity such as insulin secretion, insulin resistance and HDL-cholesterol, findings that warrant further examination in larger cohorts with data permitting genome wide association studies to be made.

## Conclusions

It is known that vitamin D insufficiency is present in about one billion people worldwide and that this high prevalence is independent of location, age and socioeconomic or cultural levels, and is mainly related to inadequate sun exposure aggravated by inadequate consumption of foods containing this vitamin. Although the relationship between vitamin D status and bone outcomes is well established [42], other studies have shown increasing evidence of strong associations between vitamin D and chronic diseases, among which is the metabolic syndrome and its component abnormalities as well T2DM and cardiovascular disease [43].

Evidence from the present study suggests an interaction between VDR gene polymorphisms and important components of MetSyn, an area where little previous work has been done. Using two common VDR polymorphisms data from this study suggest that 2 major VDR gene polymorphisms (VDR 1544410 A > G and VDR 2228570 C > T) may be linked to insulin secretion and resistance and to the degrees of reduction in serum HDL cholesterol. This evidence is in line with previous reports and suggests the need to examine data from larger cohorts, such as those where vitamin D axis genes were examined in GWAS [24], and to examine the effects of adequate vitamin D supplementation on all components of the metabolic syndrome and to determine any such effects are modulated by vitamin D axis related gene polymorphisms.

## Abbreviations

VDR: Vitamin D receptor gene; MetSyn: Metabolic syndrome; iPTH: Intact parathormone; NCEP-ATP III: National Cholesterol Educational Program - Adult Treatment Panel III; BMI: Body mass index; WC: And waist circumference; HPLC: High performance liquid chromatography; ASPCR: Allele specific polymerase chain reaction; ARM: Amplification refractory mutation; NHANES: National Health and Nutrition Examination Survey; GWAS: Genome-wide association.

## Competing interests

The authors declare that they have no competing interests.

## Authors' contributions

NJS carried out the study, data analyses and drafted the manuscript. VCG participated in the data collection and analysis. SRGFV participated in the design and coordination of the study. LAM conceived the study, and participated in its design, coordination and helped to draft the manuscript. All authors read and approved the final manuscript.

## Authors’ information

This research paper covered part of the NJS’s PhD project at the School of Public Health from University of São Paulo. VCG is a PhD student of the same University. LAM is a Professor at the School of Public Health from University of São Paulo and also the supervisor of this project. SRGFV is also Professor at this University and a collaborator of this project.
